# Communication Needs of Cancer Patients and/or Caregivers: A Critical Literature Review

**DOI:** 10.1155/2020/7432849

**Published:** 2020-05-07

**Authors:** Jieyu Li, Xingjuan Luo, Qian Cao, Yi Lin, Yinghua Xu, Qiuping Li

**Affiliations:** ^1^Wuxi Medical School, Jiangnan University, Wuxi, Jiangsu Province, China; ^2^Wuxi People's Hospital, Wuxi, Jiangsu Province, China

## Abstract

**Objective:**

Effective communication for cancer patients and/or caregivers can meet information needs, reduce caregiver burden, improve physical and mental health, and promote intimacy. The aim of this review was to identify the communication needs of cancer patients and/or caregivers and to explore their specific communication needs to guide the development of future communication interventions.

**Methods:**

Chinese and English databases were systematically searched from January 2010 to October 2019, including MEDLINE, CINAHL, PubMed, and the China Academic Journal Full-text Database. The key search terms used were “cancer” or “carcinoma” or “oncology” AND “patient” or “caregiver” or “carer” AND “communication” or “discussion” or “talk” AND “need” or “needs” or “desire.”.

**Results:**

A total of 26 articles was identified and included in this review. The findings revealed the needs of cancer patients and/or caregivers in terms of communication target, content, style, timing, and preferences. Communication targets included health professionals, peers, caregivers, and patients. Communication content included illness-related, emotional support, daily life, sexuality, death, and a way to communicate with health professionals. Communication style needed to be expressed through such things as language and communication atmosphere. Communication timing mainly referred to before treatment and approaching death. Communication preferences were related to factors such as demographics and ethnic origin.

**Conclusions:**

Cancer patients and/or caregivers have different communication needs in terms of target, content, style, and communication timing. A better understanding of the unique communication needs of patients and/or caregivers will offer health professionals detailed information on designing appropriate interventions to support cancer patients and caregivers.

## 1. Background

Evidence predicts that the incidence of cancer will increase to 22.2 million by 2030 [1]. When cancer is diagnosed, it affects both the cancer patient and the patient's entire family [2]. Throughout the cancer trajectory, open communication plays an important role for both patients and caregivers in their journey of coping with cancer as a dyad [3, 4]. Open and constructive communication can reduce cancer caregiver burden [5], promote intimacy between cancer patients and their spousal caregivers [6], and improve the physical and mental health of patients and caregivers alike [7, 8]. Lewis et al. reported that communication between cancer patients and family caregivers—including listening, talking, being respectful, and decision-making within the family—was an important part of managing family tensions and regulating coping mechanisms [9].

There is a growing recognition that the basic underpinning of comprehensive cancer treatment is communication [10, 11]. In the course of cancer treatment, there is a growing need for patients and caregivers to communicate and meet their individual information needs [12]. Taking emotional support as an example, evidence shows that both negative and positive emotions are important in communication [13]. A study found that cancer patients who expressed negative emotions and received an empathetic response from their oncologist perceived communication as being more favourable [14]. Several studies showed that positive emotional communication is associated with better patient health, both physical and mental [8, 13, 15].

Indeed, studies have verified that communication interventions tailored to the communication needs of cancer patients and/or family members brought many benefits and increased acceptance of the intervention by cancer patients and/or their caregivers, leading to improved completion rate [16, 17]. One study found that interventions tailored to four different types of family caregiver promoted communication between cancer patients and caregivers and reduced caregiver burden [16]. Another study revealed high levels of acceptance and availability of tailored online communication interventions for cancer patients and caregivers [17]. Additionally, although there was no communication intervention in this study, the findings suggested that cancer patients and caregivers from different ethnic origins had different levels of open communication. The recommendations for future communication interventions also mentioned that the characteristics of different caregiver types should be fully considered [18]. Therefore, these communication interventions and findings fully demonstrate the importance of meeting the specific communication needs of cancer patients and/or caregivers.

Some unique communication needs of cancer patients and/or caregivers have also been reported. For instance, research on cancer patient' communication needs when discussing their prognosis reported that 50% of cancer patients desired quantitative (e.g., a cancer patient's estimated survival time) and 80% wanted qualitative (e.g., whether the patient's disease can be cured) information [19]. Another study showed that not all patients wanted specific information [20]. Different types of caregivers (e.g., manager caregiver, carrier caregiver, partner caregiver, and lone caregiver) have different communication needs [16].

However, in the complex context of cancer, few reviews have fully assessed the unique communication needs of cancer patients and/or caregivers. For example, while Parker et al. reported on the communication needs of cancer patients and their caregivers, they only included patients with advanced cancer [21]. Moreover, approximately 80% of the studies included in this review were from English-speaking/Northern European countries, which limit its generalisability to countries with different cultures, such as those in Asia. There is a need to explore related research on patients in different stages of cancer and with different cultural backgrounds. Thus, the present review was conducted with the intention of filling this gap.

In addition, cancer patients and caregivers have encountered many dilemmas and challenges in communicating openly [18]. For example, one study described cancer patients' difficulties in communicating with their spouses, including a lack of agreement on the meaning of a conversation and differences in a couple's communication preferences [22]. Breast cancer patients experienced communication challenges with different information requirements, treatment decisions and needs at different stages of the cancer trajectory [11]. Communication findings for prostate cancer patients revealed that when wives avoid discussing sex in order to protect their husbands, it leads to a decline in intimacy [4, 23]. For cancer patients, discussing death is one of the most difficult challenges when the disease has progressed and they are approaching the end of life [21]. Another study found that 30% to 40% of cancer patients had moderate communication problems with their caregivers during the last week of life [24]. In summary, the above evidence suggests a need for healthcare providers to meet the communication needs of cancer patients and caregivers, shifting from a “one-size-fits-all” approach to a “personalised” approach [16, 18, 25, 26].

Consequently, the aim of this review was to identify the communication needs of cancer patients and/or caregivers and explore their personalised and specific communication needs to guide the development of future communication interventions.

## 2. Methods

### 2.1. Search and Selection Process

**Figure 1:**
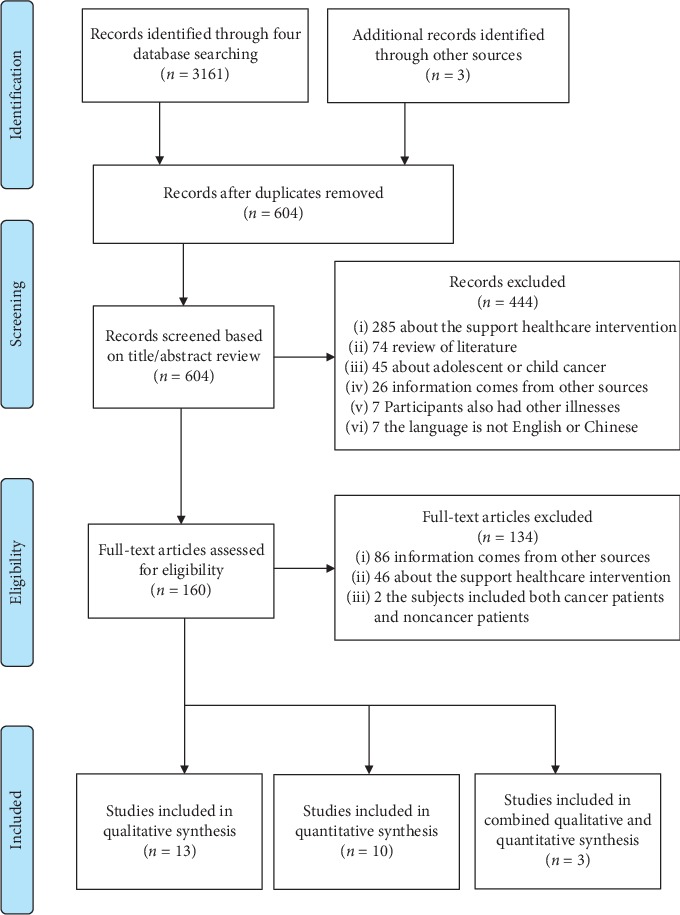
The flow diagram of literature identification.

Search terms related to the subject used “cancer” or “oncology” or “carcinoma” AND “patient” or “caregiver” or “carer” AND “communication” or “discussion” or “talk” AND “need” or “needs” or “desire.” A manual search was also performed for references cited in the articles that were included. Chinese and English databases were systematically searched from January 2010 to October 2019. With an intention to explore updated investigations in related areas, e.g., the last 10 years, the search commencement time was settled as January 2010. The following electronic databases were searched: MEDLINE, CINAHL, PubMed, and the China Academic Journal Full-text Database. The specific search strategies in this review are shown in Table S1. Article eligibility and selection were conducted in accordance with the established inclusion and exclusion criteria. EndNote was used to identify and eliminate duplicates.  Figure 1 provides a flowchart of the search and selection process.

### 2.2. Inclusion and Exclusion Criteria

All articles that were included were published in peer-reviewed journals. The specific inclusion criteria were as follows: (1) study participants were adults (≥18 years older), who were cancer patients and/or caregivers of cancer patients; (2) the research focus was the communication needs of cancer patients and/or their caregivers; (3) information came from communicating with people (e.g., communication with healthcare professionals, peers, and family members). Exclusion criteria were as follows: (1) subjects included both cancer patients and noncancer patients; (2) cancer patients and/or caregivers had other illnesses that may have resulted in research bias (e.g., HIV and severe mental illness); (3) commentaries, editorials, literature reviews, and conference proceedings were excluded.

### 2.3. Methodological Quality Appraisal

Article quality was evaluated separately by the two authors. In the event of any disagreement, a solution was discussed and agreed on by both authors. The mixed methods appraisal tool (MMAT) was used to evaluate the quality of the articles included in this review. MMAT is a reliable and effective evaluation tool that can simultaneously evaluate qualitative, quantitative, and mixed methods studies [27, 28]. In the evaluation criteria, quantitative studies were divided into quantitative randomised trials, quantitative nonrandomised trials, and quantitative descriptive studies. First, there are two screening questions to determine whether MMAT can be used for evaluation. Then, we selected the appropriate study category for each study, and finally divided the studies into three ratings: “yes,” “no,” and “cannot tell.” Although calculating total scores for quality is discouraged, five detailed scoring criteria are given for each study category to evaluate the quality of the studies that were included.

## 3. Results

### 3.1. Study Quality and Characteristics

A total of 26 articles were identified, including 10 quantitative studies, 13 qualitative studies, and three mixed studies. Of the 26 studies, 22 were conducted in Western countries, one was conducted in South Africa, while the remaining three were conducted in Asia. The study methods included semistructured interview, focus group study, and questionnaire survey. Study participants had been diagnosed with mixed cancer [29–44], breast cancer [45–49], ovarian cancer [50], cervical cancer [51], melanoma [52], and head and neck cancer [53, 54]. The details (study aims, study design, samples, cancer stage, instrument used, and key findings) can be seen in Table S2. The overall quality of the qualitative studies that were included was good. Due to unclear sample strategies/sources and low response rates, only two of the quantitative studies that were included met all criteria [40, 52]. Two mixed studies did not meet all criteria because there was no effective integrated interpretation [43, 44]. A detailed quality evaluation of the included literature is shown in Table 1.

### 3.2. Findings on Communication Needs

#### 3.2.1. Who

“Who” refers to communication targets, meaning the person that cancer patients, and family caregivers wished to communicate with. Eighteen studies covered the communication target needs of patients/caregivers. Out of this total, four studies reported the needs of caregivers [29, 31, 32, 41], while 12 reported the needs of patients [30, 34–37, 40, 44, 48–52] and two reported the needs of both patients and caregivers [42, 46]. Communication targets included healthcare professionals (*n* = 18, 69.2%), peers (*n* = 4, 15.4%), patients (*n* = 1, 3.8%), caregivers (*n* = 1, 3.8%), and others (*n* = 1, 3.8%).

Nineteen studies identified that cancer patients and/or caregivers wished to communicate with healthcare professionals. Five studies addressed the need for caregivers to communicate with health professionals [29, 31, 32, 41, 42]. Fourteen studies reported that cancer patients wanted to communicate with health professionals [30, 34–37, 40, 42, 44, 46, 48–52]. One study reported that gynecologic patients preferred to communicate with female health professionals about topics related to sexuality [37].

Four studies showed that cancer patients wanted to communicate with their peers, especially those with the same type of cancer and who had had the same experiences [46, 48, 49, 52]. In addition, Khoshnazar et al. reported that caregivers preferred to communicate with peers who had had the same caregiving experience [46].

One study showed that caregivers needed to communicate with patients [41]. Another study reported that cancer patients needed to talk openly about their illness with their family caregivers [34], while another study found that patients wanted to communicate with friends and cancer volunteers [49].

#### 3.2.2. What

“What” was defined as communication content (what cancer patients and family caregivers want to communicate). Twenty-four studies covered the communication content needs of patients/caregivers. Of these, five reported the needs of caregivers [29, 31, 32, 41, 54], 17 reported the needs of patients [30, 34–40, 43–45, 47–52], and two reported the needs of both patients and caregivers [46, 53]. Communication content includes disease-related information (*n* = 19, 73.1%), emotional support (*n* = 9, 34.6%), daily life (*n* = 3, 11.5%), sex/fertility (*n* = 3, 11.5%), death (*n* = 2, 7.7%), and how to communicate with health professionals (*n* = 2, 7.7%).

Disease-related information: three studies revealed that cancer caregivers' communication needs were all about how to reduce the disease-related symptoms (e.g., pain, breathing problems) of cancer patients as well as emotional management (e.g., how to reduce patient anxiety and fear) [32, 53, 54]. Patients' disease-related communication needs included diagnosis (e.g., interpretation of clinical test results), treatment (e.g., radiotherapy and chemotherapy, side effects, and recent advances in treatment), and rehabilitation and prognosis (e.g., how to return to normal life) [31, 32, 34–38, 40, 41, 43, 45, 47–53]. One study highlighted the need for doctors to explain advanced cancer patients' condition and possible future symptoms when moving from active to palliative care [36]. Another longitudinal study found that cancer patients had different communication content requirements at different treatment stages, while disease-related information requirements focused on the initial stage of diagnosis and treatment [34].

Emotional support: two studies showed that caregivers of patients with cancer needed professional support in providing empathy and emotional conversations [31, 53]. Seven studies showed that cancer patients had strong feelings about receiving emotional support, e.g., listening to their inner voice [46] being cared for and supported [34, 35, 46], having their situation as well as their illness understood by others [49, 53], and receiving encouragement to maintain hope for the future [36, 40]. One longitudinal study specifically noted that patients had different communication content needs at different stages of the disease [34]. In the early stages (recently diagnosed), patients expected healthcare providers to enhance their confidence in the treatment of the disease through emotional communication. In the recurrence stage, the need for emotional communication was most obvious. In the later stages, emotional support balancing hope and honesty was required [34].

Daily life: three studies have revealed what type of information patients need in order to go about their daily lives [30, 45, 53]. James-Martin et al. found that patients with breast, ovarian, and rectal cancers, who were currently undergoing or who had completed chemotherapy, needed information about a healthy and nutritious diet, reasonable exercise, and maintaining a healthy weight [30]. One study noted that metastatic breast cancer patients needed to know how to return to normal working life to maintain their quality of life [45]. Another study showed that head and neck cancer patients needed information on using the bathroom after surgery [53].

Sex/fertility: cancer patients also highlighted the need for communication about their sexual history after diagnosis. One study showed that patients receiving brachytherapy for cervical cancer needed to communicate information about their sexual history after treatment [51]. Another study showed that patients with gynecologic tumours and who were undergoing radiation therapy needed health professionals to inquire regularly about their sexual history [37]. Another study also found that cancer patients needed to discuss fertility issues after their diagnosis [44].

Death and how to communicate effectively with health professionals: one study found that caregivers of patients with advanced cancer needed health professionals to clarify their knowledge of death and to communicate the impending death of their loved one with the patient present [29]. In another study, widowers whose wives had died of cancer needed health professionals to have more specific conversations with them about death, so they could be prepared to say goodbye to their loved one [32]. Two studies found that cervical cancer patients undergoing brachytherapy [51] and caregivers of patients with head and neck cancer [54], needed information on how to communicate effectively with health professionals.

#### 3.2.3. How

“How” is denoted as communication style. Twenty studies covered the communication style needs of patients/caregivers. Of these, four reported the needs of the caregiver [29, 31, 32, 54], while 13 reported the needs of the patient [30, 34, 35, 39, 40, 44, 46–52], and three reported the needs of both patient and caregiver [33, 42, 43].

Patients and/or caregivers emphasised the need for honesty, compassion, patience, respect in communication, and need for balanced communication between truth and hope. One study found that patients needed empathy, competence, honesty, patience, and respect when communicating with their doctors [39]. Another study, conducted in India, showed that patients needed a sincere, pleasant, and positive atmosphere when communicating with health professionals, in order to build trust [46]. Two studies found that cancer patients desired communication in order to remain hopeful [33, 35]. In fact, several studies have shown that patients and/or caregivers need professionals to communicate with empathy and sincerity [32, 34, 40, 44, 49, 52]. Studies have noted that patients and/or caregivers struggled with finding a balance between authenticity and hope [32, 34].

Six studies identified the different communication (language) needs of patients and/or caregivers. In an Australian study, caregivers wanted health professionals to use direct language when talking about death, to avoid euphemisms, and to verbally acknowledge when a patient was near death [29]. Another study reported that patients wished to communicate in their mother tongue and have the opportunity to ask questions [51]. Likewise, three studies also noted that cancer patients needed easy-to-understand words based on their command of the language [34, 49, 52]. One study found that patients and caregivers needed easy-to-understand, culturally appropriate language that avoids the use of medical jargon [43].

Patients and/or caregivers emphasised the type of communication that is required. One study reported that spousal caregivers of cancer patients needed continuous, consistent, and clear communication [31]. In one study, widowed husbands whose wives had died of cancer desired a clear, truthful, and earlier prognosis [32]. In another study, cancer patients needed clear, accurate information from health professionals about their fertility, whether the results were good or bad [44].

Patients and/or caregivers emphasised the importance of delivery manner. Two studies explored caregivers' need to speak to doctors one-on-one, without the patient present [31, 33]. Two studies found that cancer patients wanted health professionals to deliver information step by step, so they had sufficient time to absorb it [33, 50]. Wen et al. found that breast cancer patients needed healthcare professionals to actively and regularly communicate with them about the disease [48]. Another study showed that patients preferred health professionals to repeatedly mention information about diet, exercise, and weight throughout their treatment, and they preferred communication in a group educational format [30]. A study on the communication preferences of Australian immigrants showed that Chinese immigrant patients as well as Anglo-Australian caregivers wanted the disclosure of more prognostic information, compared with the other immigrants [33].

Cancer patients emphasised the need for personalised communication. Three studies found that cancer patients had a strong need for personalised advice, and they were eager for personalised communication guidance from health professionals in terms of both treatment and emotional support [35, 40, 47].

Patients and/or caregivers emphasised there are different communication needs at different stages of the cancer journey. Longacre et al. explored the theory that caregivers needed a different amount of information in each time period, from patient diagnosis to the start and end of treatment [54]. At the same time, there were significant differences in caregivers' communication information needs, from diagnosis to treatment commencement (*p* < 0.001) and from treatment commencement to conclusion (*p*=0.003) [54]. One study explored how cancer patients' communication needs changed across the disease trajectory and treatment stage [34]. In the early stages of diagnosis, patients needed to hear the “bad news” from health professionals, as well as emotional support, to face their subsequent treatment. Before treatment begins, patients need to communicate with a professional to help them choose the right treatment. At the end of treatment and in the recovery stage, cancer patients need more communication with healthcare professionals in terms of emotional support. In the advanced stage of the disease, patients need more reassurance from healthcare professionals, who should balance authenticity with hope [34].

Patients and/or caregivers expressed a need for different communication channels. One study reported that older breast cancer patients preferred face-to-face or telephone communication [49]. Another study, which focused on cancer patients and caregivers who used social media to communicate, reported that 80.2% of patients and caregivers were using social media to communicate about “cancer,” with 56.0% of patients and caregivers using the Internet to ask questions after treatment and to keep in touch with their doctor, while 37.9% of patients and caregivers preferred to maintain close contact with their nurse [42].

#### 3.2.4. When

“When” refers to cancer patients' communication timing needs. There are five studies on communication time requirements [29, 30, 32, 33, 50].

Patients and/or caregivers emphasised the importance of communication timing. Four studies reported that cancer patients or caregivers needed health professionals to provide information early on. One study reported that caregivers expected health professionals to discuss the patient's prognosis early enough to enable them to say goodbye to loved ones [32]. One study reported that cancer patients needed health professionals to discuss palliative care with them as early as possible, preferably “before it is needed,” and even at any time [29]. Another study also reported that ovarian cancer patients needed information about genetic testing before undergoing surgery [50]. James-Martin et al. indicated that cancer patients needed health professionals to provide information before early treatment [30]. A study conducted in Australia by Mitchison et al. found that cancer patients and caregivers needed prognostic information when patients were close to death or when conditions became “bad” [33]. At the same time, Greek patients and caregivers expected oncologists to obtain their consent before delivering this type of information to patients, while Chinese and Arabic patients and caregiver needs were based on their emotional state and ability to “handle” receiving prognostic information [33].

### 3.3. Preferences

Eight studies compared the communication preferences of patients and/or caregivers across demographic, cross-cultural, geographic, cancer site, and medical factors [33, 36, 37, 40, 49, 50, 52, 54].

Five of the eight studies focused on demographic factors, including gender, age, work status, caregiver type, and education level [37, 40, 49, 52, 54]. A study of caregiver communication preferences found that a higher percentage of employed caregivers' desired up-to-date information about patient condition than caregivers who were not employed [54]. In addition, a higher proportion of nonspousal caregivers needed information on how to care for patients, compared with spousal/partner caregivers [54]. One study showed that female patients needed to speak with psychologists and mental health workers significantly more frequently than male patients [52]. A study conducted in Canada revealed that older breast cancer patients relied on health professionals to initiate the discussion of treatment information [49]. One study reported that patients with lower educational levels had a higher preference for supporting information [40]. Another study found that college educated patients desired more frequent communication about sexuality-related topics [37].

Two studies emphasised communication preferences when taking cross-cultural differences into consideration [33, 40]. One study found that cancer patients in China and India tended to prefer content and convenience when health professionals were delivering information, including specific information, advice on the best treatment options, and personalised counselling [40]. Another study of immigrants to Australia compared Anglo-Australian, Chinese, Arab, and Greek immigrant patient preferences for different types of communication [33]. The findings mainly revealed the following three points: first, Chinese cancer patients showed a strong preference for specific, positive information from doctors and emphasised the need for healthcare providers to seek patient consent before providing health information. Second, compared to other ethnic groups, large numbers of Anglo-Australian patients showed a strong preference for being informed when their condition deteriorated to “bad” and when they were close to death. Finally, Chinese and Arab caregivers in particular needed health professionals to communicate with them one-on-one, without the patient being present.

Four studies identified geographic factors, cancer site, and medical condition [36, 38, 50, 52]. In Umezawa et al.'s study, patients in urban hospitals preferred to suffer alone when receiving “bad” news, while patients in rural hospitals preferred to tell their families in order to decide on treatment and care together [36]. This study also found that breast cancer patients were reluctant to talk about the future, compared with patients with liver cancer, biliary tract cancer, and pancreatic cancer [36]. One study revealed that melanoma patients had higher information needs than other cancer patients, even if they reported fewer symptoms [52]. Another study found significant differences in the number of questions asked by patients who had different types of cancer [38]. Another study found that ovarian cancer patients who had undergone genetic testing were more likely to talk to geneticists, while patients who had not had genetic testing were more likely to talk to oncologists and nurses [50].

### 3.4. General Analysis of the Findings

Further analyses of the findings were conducted to compare differences according to the following four aspects: (i) differences in communication needs found in Asian studies and in those conducted in Western countries; (ii) differences in types of communication required of different health professionals; (iii) differences in communication needs between patients and caregivers (P-C) and in their interactions with health professionals (PC-HP); and (iv) differences in the communication needs of patients and family caregivers.

Table S3 shows the differences in communication needs found in Asian studies and in those conducted in Western countries. In terms of communication targets, more attention has been focused on patient-caregiver communication with health professionals in studies conducted in both Asian and Western countries. The difference is that the studies conducted in Western countries also focused on communication with others, e.g., patients [41], caregivers [34], friends, and cancer volunteers [49]. In terms of communication content, while studies conducted in both Asia and the West focused on disease-related information and emotional support, Western countries also reported on daily life [30, 45, 53], sex/fertility [37, 44], death, and how to communicate effectively with doctors [29, 32, 54]. In terms of communication style, studies conducted in both Asian and Western countries emphasised sincerity, sympathy, and personalised communication. Compared with Asian studies, Western studies found that participants seemed to prefer more direct communication.

Differences between the types of communication requirements for different health professionals are presented in Table S4. In terms of the differences in patient and/or caregiver communication needs when speaking to health professionals, women with sex-related cancer tend to be more communicative with female health professionals [37]. Patients and/or caregivers tended to discuss disease-related information with doctors [48, 50]. When discussing health information related to daily life as well as emotional support, they preferred to communicate with nurses [30, 46].

The differences in the communication needs of patients and caregivers (P-C) and in their interactions with health professionals (PC-HP) are displayed in Table S5. In terms of communication content needs, P-C share common communication themes [53]. Although both P-C and PC-HP have emotional support communication needs [31, 34–36, 39, 40, 46, 53], P-C are more dependent on caregivers during the disease recurrence phase [53]. In addition, there are more communication topics shared by patients and health professionals, e.g., daily life [30, 45, 53], sex/fertility [37, 44, 51], death, and how to communicate effectively with health professionals [29, 32, 51, 54]. In terms of communication style needs, when communicating with patients (P-C communication), caregivers focus more on the patient's status and adjust their communication style accordingly, with the intention of not causing patients to feel badly [41]. PC-HP communication entails greater communication requirements, e.g., communication language [29, 34, 43, 49, 52], delivery manner [30, 31, 33, 48, 50], and communication channel [42, 49].

Table S6 presents the differences in the communication needs between patients and family caregivers. In terms of communication targets, both patients and caregivers need to communicate with health professionals and peers. Patients and caregivers share similarities in terms of communication content, such as disease-related knowledge and emotional support. On the other hand, the differences manifested themselves in content, in that patients speak about their daily lives [30, 45, 53] and sex/fertility [37, 44, 51]. While caregivers also speak about death [29, 32], patients do not seem to want to discuss it. Patients and caregivers share similarities in terms of communication style requirements, e.g., health professional communication requires compassion, sincerity [32–35, 39, 40, 44, 46, 49, 52], appropriate language in communication [29, 34, 43, 49, 51, 52], and the appropriate communication channel [42, 49]. On the other hand, the difference is that patients need personalised communication with health professionals [35, 40, 47]. In addition, caregivers would like opportunities to communicate with health professionals alone, without the patient present [31, 33].

## 4. Discussion

In this review, the communication needs of cancer patients and/or caregivers were identified and their unique communication needs were explored. The main findings of this review focus on the following five aspects: who (communication targets), what (communication content), how (communication style), when (communication timing), and preferences (communication preferences).

These findings are generally in line with previous reviews. For instance, Parker et al. reported that the communication needs of each patient and caregiver may vary at different points in the disease process [21]. Hack et al. found that patients prefer to communicate about disease-related information with health professionals [25]. However, this review's findings also show the significance of focusing more on the emotional support provided to patients and/or caregivers. In addition, several general analyses of the findings were conducted in this review, with the intention of comparing the differences between the related aspects, e.g., differences in communication needs found in Asian studies and in those conducted in Western countries; differences in the types of communication for different health professionals; differences in the communication needs of patients and caregivers and in their interactions with health professionals, and differences in the communication needs of patients and caregivers.

### 4.1. Research Recommendations for Future Interventions

Based on the above analysis, the following discussion mainly focuses on general conclusions and/or future intervention suggestions directed to improve support for patients and their caregivers. This will be conducted from the five corresponding aspects, including who, what, how, when, and preference.

#### 4.1.1. Who: Communication Targets

The findings of this review disclosed that the most common communication target needs of patients and/or caregivers were those of health professionals and peers of patients and/or caregivers. For communication target choice, cancer patients and/or caregivers preferred having the right to choose with whom and when to communicate, as well as in what context [55]. Given that differences exist in the requirements for health professional communication type (Table S4), a coordinated and well-integrated multidisciplinary approach for health professionals is recommended when developing communication interventions, in order to meet the various communication needs of patients and/or caregivers.

#### 4.1.2. What: Communication Content

The findings of this review showed that the main content of communication needs was disease-related information and psychological support. In some cases, psychological support needs exceed disease-related information needs [39]. The findings also showed that the communication content needs of cancer patients and/or caregivers vary with the trajectory of the disease and the progression of the treatment phase. For example, more emotional support is needed during the initial diagnosis and final stage [34]. More information about the disease is needed as treatment progresses [53]. After treatment completion, support and relief of concerns about cancer recurrence are required [34]. In the final stage, comfort and encouragement are needed, and there is more consideration for balancing communication between honesty and hope [29, 32, 36]. This is a reminder that future customised communication interventions should be tailored to the disease or treatment stage of patients and/or caregivers.

#### 4.1.3. How: Communication Style

Cancer patients and/or caregivers had specific communication style preferences. Patients and caregivers generally need sincerity, compassion, and patience throughout the communication process. In terms of language, patients and/or caregivers generally need health professionals to use language that is easily understood and culturally appropriate. In terms of communication channels, in this review, the older patients preferred face-to-face and telephone communication; however, many studies have also used web-based communication interventions to achieve satisfactory results. Thus, interventions should be delivered with compassion, patience, and sincerity to patients and/or caregivers.

#### 4.1.4. When: Communication Timing

Patients and/or caregivers also have different timing requirements for information delivery. In studies on communication timing, most patients and/or caregivers preferred health professionals to communicate with them prior to treatment and then to communicate with them at every stage thereafter. This may be due to the fact that patients and/or caregivers wanted more time to consider and choose when they would be fully informed about the disease and treatment. Therefore, it would be safe to suggest that communication interventions be provided as early as possible only if it was a patient's and/or caregiver's preference.

#### 4.1.5. Preferences: Communication Preferences

Communication preferences of cancer patients and/or caregivers were identified in this review as being related to multiple factors, including demographics, culture, geography, cancer site, and medical condition. Whether cancer site has an effect on communication needs remains inconclusive. Studies in this review have shown the effects of different cancer sites on information needs and the number of questions asked [38, 52]. However, one study showed no significant differences in communication preferences among patients with different cancer types [20]. This is a reminder that future research is warranted, to further explore the communication needs of patients with different types of cancer. Nevertheless, it is recommended that factors such as demographics, culture, geography, cancer site, and medical condition be fully considered when developing interventions to address the specific communication needs of patients and/or caregivers.

In summary, when developing communication interventions, health professionals should fully consider the aforementioned five aspects, namely, who, what, how, when, and preferences. Only in this way can related tailored communication interventions be developed and delivered to address the specific communication needs of patients and/or caregivers.

Correspondingly, it is suggested that future interventions be tailored to the communication needs and preferences of specific cancer patients and/or caregivers [16, 17]. In terms of communication, research has identified four specific caregiver types: manager caregivers, carrier caregivers, partner caregivers, and lone caregivers [16, 26, 41]. Manager caregivers and carrier caregivers, who assume the role of decision maker for doctors and patients, limit the variety of conversation topics, avoid talking about death, and rarely communicate with patients [26]. In fact, they also need to communicate with other family members and on a broader range of topics [16]. Partner caregivers participate in decision making with patients; this type of caregiver needs to communicate more with health professionals to obtain information about the disease [16]. Lone caregivers neglect emotional communication and should increase caregiver-patient communication on emotion-laden topics [26].

Tailored communication for cancer patients and/or caregivers helps improve both parties' comfort level and better meets their communication needs [46, 54]. In addition, when meeting the communication needs of patients and/or caregivers, emotional needs should also be taken into account. An empathetic response and supportive communication can enhance the communication effect [25]. What patients require is genuine empathy from professionals, as opposed to generalised answers [56]. One study found that a lack of professionalism and adequate communication on the part of health professionals led to feelings of uncertainty in spousal caregivers, who sometimes only needed a simple greeting from health professionals who were on the ward every day [31].

In addition, before communicating with cancer patients and/or caregivers, healthcare professionals should fully consider and evaluate the potentially fragile emotional state of patients and/or caregivers and use words that ensure effective communication, while keeping patients and/or caregivers hopeful. In fact, a study on communication interventions applied the emotional disclosure scale to measure level of expression on cancer-related issues [57]. Truthfully, regardless of who it is that cancer patients and/or caregivers are communicating with, the ultimate goal is to achieve mutual understanding [58]. Overall, each cancer patient and/or caregiver has their own individual needs and desires for individualised communication.

### 4.2. Study Gaps Identified

Of the 26 studies included in the review, only three were conducted in Asia. While a variety of cancer patient and/or caregiver communication needs were explored in this study, there was no systematic or comprehensive assessment of their communication needs. Although the studies that were included described many of the communication needs of patients and/or caregivers, few addressed the communication needs of patient-caregiver dyads.

### 4.3. Limitations of This Review

Several limitations in this review must be acknowledged. First, due to the significant results of the published studies, publication bias was unavoidable in the literature review process. Second, the communication needs of cancer patients and/or caregivers with a multicultural background are lacking.

### 4.4. Clinical Implications

Based on the results of this review, the following are clinical implications for health professionals when communicating with patients and/or caregivers.

Health professionals should assess the content, style, time, and preference needs of cancer patients and/or caregivers in order to summarise their unique needs before communicating with them. For example, what is needed most in the current stage of communication? What is the patient's communication style and cultural preferences? What is the patient's and/or caregiver's current ability to “handle” information? and what is their emotional state? Above all, health professionals should communicate with patients and/or caregivers with compassion and not simply provide general answers.

In addition, there are clinical implications for future communication interventions to assess the needs of both patients and caregivers. In order for clinical staff to assess the communication needs of patients and caregivers more systematically and comprehensively, future studies could develop corresponding communication questionnaires or even scales. A communication needs questionnaire or scale should be designed to take the cultural background of the patient and/or caregiver into account.

Furthermore, the primary informal caregiver for cancer patients is the spouse [59, 60], and the unique communication needs of spousal caregivers and cancer patients should be explored in future research.

## 5. Conclusion

This review summarises the specific communication needs of cancer patients and/or caregivers, including communication target selection, differences in communication content, and dynamically changing communication needs and preferences in other situations. In addition, study gaps, review limitations, and clinical implications were also discussed. In summary, this better understanding of the communication needs of patients and/or caregivers allows health professionals to design interventions to support cancer patients and caregivers, based on the personalised communication needs of cancer patients and/or caregivers.

## Figures and Tables

**Table 1:** Quality assessment table according to MMAT.

*Qualitative*	*1.1. Is the qualitative approach appropriate to answer the research question?*	*1.2. Are the qualitative data collection methods adequate to address the research question?*	*1.3. Are the findings adequately derived from the data?*	*1.4. Is the interpretation of results sufficiently substantiated by data?*	*1.5. Is there coherence between qualitative data sources, collection, analysis, and interpretation?*
Collins et al. [29]	Yes	Yes	Yes	Yes	Yes
Danesh et al. [45]	Yes	Yes	Yes	Yes	Yes
Gleeson et al. [50]	Yes	Yes	Yes	Yes	Yes
James-Martin et al. [30]	Yes	Yes	Yes	Yes	Yes
Khoshnazar et al. [46]	Yes	Yes	Yes	Yes	Yes
Laursen et al. [31]	Yes	Yes	Yes	Yes	Yes
Long et al. [51]	Yes	Yes	Yes	Yes	Yes
Mitchison et al. [33]	Yes	Yes	Yes	Yes	Yes
Thorne et al. [34]	Yes	Yes	Yes	Yes	Yes
Wen et al. [48]	Yes	Yes	Yes	Yes	Yes
Wittenberg et al. [41]	Yes	Yes	Yes	Yes	Yes
Wong et al. [49]	Yes	Yes	Yes	Yes	Yes
Yi et al. [47]	No	Yes	Yes	Yes	Yes

*Quantitative descriptive*	*4.1. Is the sampling strategy relevant to address the research question?*	*4.2. Is the sample representative of the target population?*	*4.3. Are the measurements appropriate?*	*4.4. Is the risk of nonresponse bias low?*	*4.5. Is the statistical analysis appropriate to answer the research question?*

Ahamad et al. [38]	No	Yes	Yes	Cannot tell	Yes
Bartholomäus et al. [39]	Cannot tell	Cannot tell	Yes	Yes	Yes
Braun et al. [42]	No	Yes	Yes	Yes	Yes
Chapman et al. [37]	Yes	Cannot tell	Yes	Yes	Yes
Eng et al. [40]	Yes	Yes	Yes	Yes	Yes
Longacre et al. [54]	Yes	Cannot tell	Yes	Yes	Yes
Passalacqua et al. [52]	Yes	Yes	Yes	Yes	Yes
Rodriguez et al. [53]	Yes	Cannot tell	Yes	Yes	Yes
Umezawa et al. [36]	Yes	Yes	Yes	No	Yes
van Weert et al. [35]	Cannot tell	Yes	Yes	No	Yes

*Mixed methods*	*5.1. Is there an adequate rationale for using a mixed methods design to address the research question?*	*5.2. Are the different components of the study effectively integrated to answer the research question?*	*5.3. Are the outputs of the integration of qualitative and quantitative components adequately interpreted?*	*5.4 Are divergences and inconsistencies between quantitative and qualitative results adequately addressed?*	*5.5 Do the different components of the study adhere to the quality criteria of each tradition of the methods involved?*

Alananzeh et al. [43]	Yes	Yes	Yes	Yes	Yes
Park et al. [32]	Yes	No	Yes	Yes	Yes
Ussher et al. [44]	Yes	No	Yes	Yes	Yes

## Supplementary Materials

Supplementary Materials Table S1: search strategies; Table S2: communication needs of cancer patients and/or caregivers; Table S3: differences in communication needs found in Asian studies and in those conducted in Western countries; Table S4: differences between the types of communication requirements for different health professionals; Table S5: differences in the communication needs of patients and caregivers (P-C) and in their interactions with health professionals (PC-HP); Table S6: differences in the communication needs between patients and family caregivers.
