# Supplementary material for: Communication Needs of Cancer Patients and/or Caregivers: A Critical Literature Review
Source: J Oncol. 2020 May 7;2020:7432849. doi: 10.1155/2020/7432849 (PMC7229568; doi:10.1155/2020/7432849)
Supplement: Supplementary Materials — Table S1: search strategies; Table S2: communication needs of cancer patients and/or caregivers; Table S3: differences in communication needs found in Asian studies and in those conducted in Western countries; Table S4: differences between the types of communication requirements for different health professionals; Table S5: differences in the communication needs of patients and caregivers (P-C) and in their interactions with health professionals (PC-HP); Table S6: differences in the communication needs between patients and family caregivers. [file 7432849.f1.pdf]

Table S1 Search Strategies

Pubmed	
#1	Search ((cancer [MeSH Terms]) OR oncology [Title/Abstract]) OR carcinoma [Title/Abstract] Filters: Publication date from 2010/01/01 to 2019/10/31
#2	Search ((patient [MeSH Terms]) OR caregiver [Title/Abstract]) OR carer [Title/Abstract] Filters: Publication date from 2010/01/01 to 2019/10/31
#3	Search ((communication [MeSH Terms]) OR discussion [Title/Abstract]) OR talk [Title/Abstract] Filters: Publication date from 2010/01/01 to 2019/10/31
#4	Search ((need [MeSH Terms]) OR needs [Title/Abstract]) OR desire [Title/Abstract] Filters: Publication date from 2010/01/01 to 2019/10/31
#5	#1 AND #2 AND #3 AND #4
CINAHL	
#1	TI cancer OR TI oncology OR TI carcinoma
#2	AB cancer OR AB oncology OR AB carcinoma
#3	TI patient OR TI caregiver OR TI carer
#4	AB patient OR AB caregiver OR AB carer
#5	TI communication OR TI discussion OR TI talk
#6	AB communication OR AB discussion OR AB talk
#7	TI need OR TI needs OR TI desire
#8	AB need OR AB needs OR AB desire
#9	#1 OR #2
#10	#3 OR #4
#11	#5 OR #6
#12	#7 OR #8
#13	#9 AND #10 AND #11 AND #12
Medline	The same as the CINAHL database search strategy

Table S2 Communication needs of cancer patients and/or caregivers

Quantitative studies

**Authors (country of the study)**	**Aims**	**SD**	**Samples/ cancer stage**	**Instrument Used**	**Significant Findings**	
					**Patient**	**Caregiver**
Ahamad et al.<br>2019<br>(USA)<br>（38）	To estimate the quantity, nature, and variation of their questions when they were invited to ask by their oncologist	C	-396 cancer patients<br>**cancer stage**: all phases	-A checklist of topics	**What**<br>-Disease-related information (comprised 36.7% of all questions): diagnosis, rehabilitation, chemotherapy and drug information;<br>-Radiotherapy (comprised 56.9% of all questions): details of radiotherapy and management of acute side effects.	
Bartholomäus et al.<br>2019<br>(Germany)<br>(39)	To understand patients' needs for virtue and physician behavior	C	-186 cancer patients<br>**cancer stage**: not mention	-The 5-items virtues need questionnaire	**What**<br>-Needed doctors to take the time to listen to them talking about their symptoms or doubts about the disease or treatment;<br>**How**<br>-Competence, Honesty, Respect, Patience and Compassion when communicating with doctors;<br>-Expected doctors to treat them equally	

					in the process of communication.
Braun et al. 2019 (Germany) (42)	To evaluate cancer patients' need for information, their communication and usage of social media	C	-370 cancer patients<br>-28 caregivers<br>**cancer stage:** not mention	-The 6-items standard questionnaire	**Who**<br>-Most of the participants needed to get information from doctor (85.9%);<br>-56.0% of the participants hoped to keep in close contact with doctors, and 37.9% of the participants hoped to keep in close contact with nurses.<br>**How**<br>−80.2% of the participants got in contact with other people about the topic 'cancer' via social media;<br>-63.1% of the participants asked questions of other patients through the Internet, 56.0% of the participants asked questions of experts through the Internet.
Chapman et al. 2019 (USA) （37）	To assess the beliefs/attitudes of women receiving follow-up treatment for gynecologic cancer in the radiation oncology department toward providers' sexual history taking	C	-75 women received follow-up treatment for gynecologic cancer in the radiation oncology department<br>**cancer stage:** not mention	-The 19-items Female Sexual Function Index (FSFI)	**Who**<br>-62.7 % respondents needed to communicate sexual history issues with female health professionals;<br>**What**<br>-62.7% of patients needed that medical providers ask about their sexual history on a regular basis;<br>**Preferences**<br>- Patients with some college degree, e.g., associate's degree, bachelor's degree and postgraduate degree, need a regular discussion of their sexual history.

Eng et al. 2012 (Malaysian) (40)	To describe the preferences of Malaysian cancer patients regarding the communication of bad news	C	-200 cancer patients<br>**cancer stage**: all phases	-The Malay language version of the Measure of Patients' Preferences (MPP-BM)	**Who**<br>-To communicate with health professionals;<br>**What**<br>-The best treatment options, details about medical test results and the latest scientific research;<br>-Emotional support to give them hope;<br>**How**<br>-To tell them in person, in detail and honestly;<br>**Preferences**<br>-Compared with Malays, Chinese and Indian respondents preferred "content and convenience";<br>-With lower educational status had a higher preference for "Structural and Informational Support".	
Longacre et al. 2015 (USA) （54）	To explore head and neck cancer caregivers' informational needs including those related to interacting in the medical context as a caregiver and	C	-59 caregivers<br>**cancer stage:** not mention	-Abbreviated form of the Cancer Caregiver Needs Checklist<br>-Caregiver information source preference was assessed using		**What**<br>-74.6% caregivers reported having high information need at diagnosis related to interacting as a caregiver;<br>- To reduce a patient's pain or distress at diagnosis (66.1%), treatment start (74.6%), and at

	meeting patient needs and preferences for obtaining caregiving information and information source preference	survey questions from the National Alliance for Caregiving and AARP		treatment end (44.1%);<br>-How to obtain the latest information about the patient's condition and how to play an active role in care decisions during the patient's diagnosis;<br>-Need information on how to talk to a doctor or nurse;<br>**How**<br>-Needed different amounts of relevant information at each point of treatment, from diagnosis (74.6%) to the beginning of treatment(66.1%）to the end of treatment(50.8%);<br>-Communication needs differed significantly from diagnosis to treatment end (p < .001) and from treatment start to treatment end (p = .003) but not from diagnosis to treatment start;<br>**Preferences**<br>-Who were employed have higher information needs than

						caregivers who were not employed and who were non-spouse have higher information needs than spouse/partner caregivers.
Passalacqua et al. 2012<br>(Italy)<br>（52）	To evaluate the information needs of patients with advanced melanoma compared to patients with other malignancies	C	-221 cancer patients<br>**cancer stage**: all phases	-The 23-items information need questionnaire	**Who**<br>-To talk to people who have the same experience;<br>-To communicate with health professionals;<br>**What**<br>**-**About diagnosis, treatment, testing and prognosis, a higher need for information compared to patients with other cancers, even if Melanoma patients reported fewer symptoms;<br>**How**<br>**-**Melanoma patients needed more sincere and easily understood conversations from health professionals compared to patients with other cancers<br>**Preferences**<br>-Women expressed the need to speak with a psychologist and with a spiritual assistant significantly more frequently	

					than men;<br>-Female melanoma patients needed more frequent reassurance from a nurse than other cancer patients.	
Rodriguez et al. 2010 (USA) （53）	To identify communication needs for nonspeaking patients with head and neck cancer during their acute care hospitalization after surgery and explore whether family caregivers' and nurses' perceptions of communication needs differed from those identified by cancer patients.	C	-11 patients who were nonspeaking after head and neck cancer surgery,8 family caregivers<br>**cancer stage:** not mention	-The 5-items communication needs survey questionnaire	**What**<br>-Immediate needs: about disease treatment and daily living communication needs, including pain and postoperative breathing problems;<br>-Psychosocial needs: hoped to alleviate fear and loneliness through communication.	**What**<br>-Psychosocial needs: needed to know how the patient is feeling and how to relieve their negative emotions;<br>-Immediate needs: how to instantly relieve patients' treatment-related problems, including breathing problems and pain.
Umezawa et al. 2015 Japan （36）	To clarify the communication preferences of patients with advanced cancer regarding discussions about ending anticancer treatment	C	-106 cancer patients<br>**cancer stage**: all phases	-The 57-items information need questionnaire	**Who**<br>-To communicate with doctors;<br>**What**<br>-To explain changes in their condition and future symptoms as they switch from cancer treatment to palliative care;	

	and transitioning to palliative care				-Emotional support to maintain hope for the future;<br>**Preferences**<br>-Younger patients preferred the provision of a satisfactory amount of detail information;<br>-When receiving 'bad' news, patients in urban hospitals tended to suffer alone, while patients in rural hospitals first told their families and communicate treatment;<br>- Breast cancer patients were reluctant to discuss palliative care symptoms and information about the future, while patients with liver, gallbladder and pancreatic cancer who have children were more likely to be told about life expectancy and to be mentally prepared to maintain hope for the future;<br>-Patients who had been diagnosed more recently preferred their physician to behave with empathic paternalism.
van Weert et al. 2013 (The	To understand the information and communication	C	-155 older cancer patients<br>**cancer stage**: all	-The Groningen frailty indicator (GFI)	**Who**<br>-To communicate with health professionals;

Netherlands) （35）	needs of older cancer patients at the start of chemotherapy treatment	phases	-The 66-items of the QUOTE^{chemo}-Importance questionnaire for elderly cancer patients	**What**<br>-Treatment-related information, rehabilitation information, affective communication and realistic expectations;<br>**How**<br>-To get tailored communication.

Abbreviations: SD, Study design; C, Cross-sectional study; QUOTE, ^{chemo} Quality of care through the chemotherapy treatment patients' eyes.

<u>**Qualitative studies**</u>

Authors (country of the study)	Aims	SD	Informants	Significant Findings	
				**Patient**	**Caregiver**
Collins et al. 2018 (Australia) (29)	To explore caregiver perspectives on communication about death, dying and the introduction to palliative care	C	-25 caregivers for cancer patients<br>**cancer stage**: advanced cancer		**Who**<br>-To communicate with health professionals;<br>**What**<br>-To clarify how much they wish to know about the dying process;<br>- To tell them that the patient is going to die proactively;<br>- Needed the medical team to be able to communicate the impending death of their loved ones with patients present;<br>**How**<br>-To use the direct language 'death' to

					communicate and avoided euphemistic language, but avoided direct communication at the end of life;<br>-Oral confirmation of family members by health professionals when death approaches;<br>-To separate the educational task of palliative care practice from the discussion of patient referrals;<br>**When**<br>-To discuss as early as possible, 'before it is needed';<br>-To discuss at all times.
Danesh et al. 2013 (USA) (45)	To explore what questions patients with metastatic breast cancer ask their oncologists	D	-59 cancer patients<br>**cancer stage**: metastatic cancer	**What**<br>-Disease-related information: diagnosis, symptom management, clinical trials and palliative care;<br>-About quality of life and daily activities.	
Gleeson et al. 2013 (Australia) (50)	To identify ovarian cancer patients' information and communication preferences about treatment-focused genetic testing(TFGT) in the ovarian cancer context	SI	- 22 women diagnosed with ovarian cancer who had either advanced disease and had previously undergone TFGT (n = 12) or had	**Who**<br>-Needed geneticist, oncologist or nurse to deliver information, but preferred their medical oncologist to deliver the information;<br>**What**<br>-About the implications of TFGT for their family, the latest drugs for ovarian cancer and the side effects of treatment;<br>**How**	

			been diagnosed in the previous 6–20 weeks with ovarian cancer and had not undergone TFGT (n = 10)<br>**cancer stage**: all phases	-Desired verbal information in the first instance;<br>-To give them information step by step;<br>**When**<br>-About TFGT early, before ovarian surgery;<br>**Preferences**<br>-Those who have undergone genetic testing preferred to communicate with geneticists for information, while those who do not have a genetic test were more likely to communicate with their oncologist or nurse for information.	
James-Martin et al. 2014 (Australia)（30）	To assess the information needs of patients undergoing curative chemotherapy regarding diet, exercise and weight management for the purpose of developing weight management resources	FG	-15 cancer patients and survivors<br>**cancer stage**: all phases	**Who**<br>-Consider nurses as the preferred source of communication information;<br>**What**<br>-Detailed and specific information about diet, exercise and weight management;<br>**How**<br>-To mention information repeatedly such as exercise diet throughout treatment;<br>-Preferred group education to communicate;<br>**When**<br>-Needed information at the beginning of early treatment.	
Khoshnazar et al.	To examine the perception of women	SI	- 9 women with breast cancer, 10	**Who**<br>-To communicate with health professionals,	**Who**<br>-To communicate with people who have the

2016<br>(Iran)<br>(46)	with breast cancer in terms of their communication needs		health-care professionals, and one family caregiver<br>**cancer stage**: all phases	particularly nurses;<br>-To communicate with peers;<br>**What**<br>-Emotional support and compassionate care;<br>**How**<br>-A sincere, positive and happy chat atmosphere with health professionals.	same experience.
Laursen et al.<br>2015<br>(Denmark)<br>（31）	To identify the needs of spouses, and develop professional relations and well-planned communication with health professionals	FG	-11 spouses of cancer patients<br>**cancer stage**: not mention		**Who**<br>-To communicate with health professional;<br>**What**<br>-Needed support and understanding from communication;<br>-Needed to communicate their experiences and suffering with health professionals;<br>**How**<br>-Continuous, clear, consistent, professional and well-planned communication.
Long et al.<br>2016<br>(South Africa)<br>（51）	To identify informational needs of South African women receiving intracavitary brachytherapy for locally advanced cervical cancer as part of a process to develop guidelines for	P	-28 patients received brachytherapy for cervical cancer<br>**cancer stage**: not mention	**Who**<br>**-**To communicate with their doctors;<br>**What**<br>- How to communicate with health professionals;<br>**-** Disease and treatment: instructions on brachytherapy procedures, management of side effects, preparation before treatment, and specific dates of treatment;	

	quality<br>patient-centred care			-The possibility of sexual intercourse and the possibilities of subsequent childbearing;<br>-Needed the opportunity to ask questions;<br>**How**<br>**-** Needed easy-to-understand information in their own language.	
Mitchison et al. 2012 (Australia) （33）	To explore communication preferences for prognostic information in English speaking and migrant patients and their families	SI	-73 cancer patients<br>-66 caregivers<br>**cancer stage**: new diagnosis	**How**<br>-31 patients expressed a preference for openly provide all details about patients' prognoses;<br>-24 patients expressed a desire for non-disclosure of prognosis;<br>-Needed positive information to keep them hopeful;<br>-Needed to give them information step by step;<br>**When**<br>-8 patients wanted prognosis information when conditions were 'worse' and the patient was close to death;<br>-Emphasized that oncologists must ask patients' permission before giving them information;<br>-Based on their emotional state and 'handle' ability to provide prognostic information to them;<br>**Preferences**<br>-Compared with other immigrant patients in	**How**<br>**-**17 relatives expressed a preference for oncologists to openly provide all details about patients' prognoses, among them, compared with other countries, Anglo Australians were more willing to fully disclose;<br>-30 relatives expressed a desire for non-disclosure of prognosis;<br>-Needed positive information to keep patients hopeful;<br>-Needed to provide information step by step;<br>-Needed to consult with doctors alone, without the patient present;<br>**When**<br>-7 relatives wanted prognosis information when conditions became 'worse' and patients were close to death;<br>-14 relatives emphasized that oncologists must ask patients' permission before giving

				Australia, Chinese patients showed the strongest communication preferences in terms of the doctors providing detailed and positive information, as well as the patients' consent before providing information;<br>-Anglo Australian patients did not want health professionals to disclose their prognosis and wished to be informed when they were closed to death in comparison to other ethnic patient groups.	patients information;<br>-18 relatives needed doctors to determine whether to provide prognostic information, based on relative's emotional state, age and ability to process information;<br>**Preferences**<br>**-**Chinese caregivers were more likely to consult doctor alone than migrants from other countries.
Thorne et al. 2014 (Canada)（34）	To explore how cancer patients' communication needs and preferences change as their disease progresses	L	- 125 cancer patients<br>**cancer stage**: all phases	**Who**<br>**-**To communicate with health professionals and cancer care providers;<br>**What**<br>-During diagnosis, expected personalized advice and empathy;<br>-In the treatment phase, needed communicate to help them make treatment decisions;<br>-At the end of the treatment phase, needed to talk about illness and emotional support;<br>-The recurrence phase required active emotional support from cancer care providers;<br>-Advanced disease, the need for communication was to strike a balance between hope and honesty;<br>**How**	

				-Needed easy-to-understand words in communication.	
Wen et al. 2014 (USA) (48)	To examine the experiences of Chinese American breast cancer survivors to better understand their information and communication needs and their preferences for survivorship care planning	SI	-16 Chinese American breast cancer survivors<br>**cancer stage**: all phases	**Who**<br>-To communicate with peers;<br>-To communicate with health professionals of the same cultural background;<br>**What**<br>-About diagnosis, treatment options, and the common side effects of treatment, recurrence, lifestyle changes;<br>-Evidence-based, culturally and linguistically appropriate health information;<br>**How**<br>-To initiate and regularly discuss treatment with them.	
Wittenberg et al. 2017 (USA) (41)	To describe a family caregiver communication typology and demonstrate identifiable communication challenges among four caregiver types: Manager, Carrier, Partner, and Lone	ID	-4 caregivers<br>**cancer stage**: not mention		**Who**<br>-To communicate with patients and health professionals;<br>**What**<br>-Manager caregiver and carrier caregiver needed to discuss broader life topics, treatment side effects and goals of treatment;<br>-Lone caregiver needed to discuss the patient's illness information.<br>**How**<br>-Partner caregiver needed to openly discuss

					caregiver stress to minimize caregiver stress;
Wong et al. 2011 (Canada) (49)	To investigate the information needs and unique illness experiences of older women with early stage breast cancer	FG and OI	-16 older patients with early breast cancer<br>**Cancer stage:** early stage	**Who**<br>-To communicate with health professionals, peers, friends and cancer volunteers;<br>**What**<br>-Interpret and help them understand medical terminology and treatment options;<br>-Emotional support;<br>**How**<br>-To communicate with empathy;<br>-To speak at the same language level as patients;<br>**Preferences**<br>-Older patients preferred face-to-face and telephone communication about treatment;<br>-Older patients preferred to rely on health professionals to open up space to discuss treatment information.	
Yi et al. 2015 (USA) (47)	To identify barriers and facilitators to patient-provider communication when discussing breast cancer risk to aid in the development of decision support tools	FG	-34 breast cancer patients<br>**cancer stage**: not mention	**What**<br>-Needed information about the disease;<br>**How**<br>-Needed personalized advice and guidance.	

Abbreviations: SD, Study design; D, Descriptive; P, Phenomenological Perspective; L, Longitudinal Study; SI, Semi-structured Interview; DI, Depth Interview; FG, Focus group; OI, One-on-One Interview; ID, Interpretive description; CP, cancer patients; TFGT, treatment-focused genetic testing.

Combined Quantitative and Qualitative Studies

**Authors (country of the study)**	**Aims**	**SD**	**Sample Size (n)/cancer stage**	**Instrument Used**	**Significant Findings**
Alananzeh et al. 2019 (Australia) （43）	To identify the information needs and information sources among Arab cancer survivors and their caregivers	Survey and interviews	-143 Arab cancer survivors/caregiver **cancer stage**: all phases	-The 34-items supportive care needs survey (SCNS-34) -A 13-item unpublished tool	**What** -Cancer survivors expressed greater need for information about managing their illness, receiving explanations about tests, and the benefits and side-effects of treatments before making choices than Jordanian cancer survivors; -To receive culturally appropriate information; **How** -Easily digestible format and in their language of choice without medical jargon; -Many participants preferred face-to-face oral communication over written forms of communication; **When** -To deliver information at the right time based on condition;
Park et al. 2015 (USA)	To explore end-of-life prognostic	Survey and interviews	- 244 men whose wives died of cancer and were the fathers	-The open-ended survey questions about prognostic	**Who** -To communicate with health professionals; **What**

（32）	communication experiences reported by bereaved fathers whose wives died from cancer		of their children.	communication<br>-Semi-structured interview	-About wives' disease courses, death processes, potential treatment complications, and palliative and hospice care;<br>**How**<br>-To communicate with empathy;<br>-To communicate clearly and honestly about the prognosis;<br>**When**<br>-To communicate the prognosis earlier so that he and the child would have time to say goodbye to his wife.
Ussher et al. 2018 (Australia) (44)	To examine the construction and subjective experience of communication with health professionals about fertility in the context of cancer	Survey and interviews	-878 cancer patients<br>**cancer stage**: all phases	-The closed and open ended survey questions<br>-Semi-structured interview	**Who**<br>-To communicate with health professionals;<br>**What**<br>-To discuss fertility since diagnosis;<br>**How**<br>- To be honest about their fertility since cancer diagnosis;<br>-To be informed clearly and accurately;<br>-To communicate with compassion.

Abbreviations: SD, Study design.

Table S3 Differences in communication needs found in Asian studies and in those conducted in Western countries

Category	Asia(n=3)	Western(n=22)
	Iran (46*), Japan (36), Malaysian (40)	USA (37,38,54,53,48,41,47,32,45), Italy(52), Germany (39,42), The Netherlands (35), Australia (29,50,30,33,43,44), Denmark (31), Canada (34,49)
**Who**	- Healthcare professionals (40,36,46)<br>- Peers(46)	-Healthcare professionals (42,37,52,35,29,50,30,31,34,48,41,49,32,44)<br>- Peers (52,48,49)<br>- Patients (41)<br>-Caregivers (34)<br>-Others(49)
**What**	- Disease-related information (40,36)<br>- Emotional support (40,36,46)	-Disease-related information (38,54,52,53,35,45,50,31,34,48, 41,49,47,43,32)<br>-Emotional support (39,53,35,31,34,49)<br>- Daily life (53,45,30)<br>- Sex/fertility (37,44)<br>- Death (29,32)<br>- How to communicate effectively with health professionals(54)
**How**	- Sincere, pleasant, and positive atmosphere (46); empathy, competence, honesty, patience, and respect (40)<br>- Personalised communication (40)	- Empathy, competence, honesty, patience, and respect (32,34,39,44,49,52); remain hopeful (33,35); balance authenticity and hope (32,34)<br>- Direct language (29); easy-to-understand words (34,49,52); avoid medical jargon (43)<br>- Type of communication: continuous, consistent, and clear (31); clear, truthful, and earlier prognosis (32); accurate, whether the results were good or bad (44)<br>- Delivery manner: without the patient present (31,33); step by step (33,50);

		actively and regularly communicate (48); repeatedly mention (30);<br>- Personalized communication (35,47)<br>- Different needs at different stages of cancer (54,34)<br>- Communication channels: face-to-face or telephone(49); social media, Internet(42)
**When**		- Provide information early on (29,30,32,50)<br>- Close to death (33)<br>- Obtain consent (33)
**Preference**	-Demographic factors: education level (40);<br>-Cross-cultural differences (40)<br>-Geographic factors(36); cancer site (36)	- Demographic factors: work status (54); caregiver type (54); gender (52); age (49); education level (37)<br>- Cross-cultural differences (33)<br>- Cancer site(52,38); medical condition (50)

*The number in brackets indicates the related reference literature number cited in the manuscript.

Table S4 Differences between the types of communication requirements for different health professionals

Category	doctors	nurses	cancer volunteers
**Cancer type**	gynecologic malignancies (37*)<br>ovarian cancer (50)<br>breast cancer (48)	breast, ovarian, and rectal cancer (30)<br>breast cancer (46)	breast Cancer (49)
**Who**	-Female provider (37)<br>-Preferred their medical oncologist to deliver the information (50)<br>-To communicate with health professionals of the same cultural background (48)	-Consider nurses as the preferred source of communication information (30)<br>- To communicate with health professionals, particularly nurses (46)	
**What**	-Sexual health (37)<br>- About the implications of TFGT for their family, the latest drugs for ovarian cancer and the side effects of treatment (50)<br>-Evidence-based, culturally and linguistically appropriate health information(48)	-Detailed and specific information about diet, exercise and weight management (30)<br>-emotional support and compassionate care(46)	
**How**	-Desired verbal information in the first instance; to give them information step by step (50)<br>-To initiate and regularly discuss treatment with them (48)	-To mention information repeatedly such as exercise diet throughout treatment; preferred group education to communicate(30)	- Face-to-face or telephone (49)
**When**		- Needed information at the beginning of early treatment (30)	

TFGT: treatment-focused genetic testing

*The number in brackets indicates the related reference literature number cited in the manuscript.

Table S5 Differences in the communication needs of patients and caregivers (P-C) and in their interactions with health professionals (PC-HP)

Category	P-C	PC-HP
**What**	-Patients are more emotionally dependent on their caregivers during the recurrence stage (34*)<br>-Manager caregiver and carrier caregiver needed to discuss broader life topics, treatment side effects and goals of treatment (41)	- Disease-related information (31,32,34-38,40,41,43,45,47-54)<br>- Emotional support (31,53,46,39,34,35,36,40,49)<br>- Daily life (30,45,53)<br>- Sex/fertility (37,44,51)<br>- Death and how to communicate effectively with health professionals(29,32,51,54)
**How**	- Partner caregiver needed to openly discuss caregiver stress to minimize caregiver stress(41)	- Empathy, competence, honesty, patience, and respect (32,34,39,44,49,52); remain hopeful (33,35); balance authenticity and hope (32,34)<br>- Direct language (29); easy-to-understand words (34,49,52);<br>avoid medical jargon (43); mother tongue (51)<br>- Type of communication: continuous, consistent, and clear (31);<br>clear, truthful, and earlier prognosis (32); accurate, whether the results were good or bad (44)<br>- Delivery manner: without the patient present (31,33); step by step (33,50); actively and regularly communicate (48); repeatedly mention (30)<br>- Personalised communication (35,40,47)<br>- Different needs at different stages of cancer (54,34)<br>- Communication channels: face-to-face or telephone(49); social media, Internet (42)

*The number in brackets indicates the related reference literature number cited in the manuscript.

Table S6 Differences in the communication needs between patients and family caregivers

Category	Patients		Caregivers
**Who**	-Health professional (30,34,35,36,37,40,42,44,46,48-52*)		-Health professional (29,31,32,41,42)
	-Peers (46,48,49,52)		-Those have the same caregiving experience (46)
	-Friend and cancer volunteer (49)		
**What**	-Disease-related information (31,32,34-38,40,41,43, 45,47-53)		-Disease-related information (32,53,54)
	-Emotional support (46,39,34,35,46,49,53,36,40)		-Emotional support (31,53)
	-Daily life (30,45,53)		-Death (29,32)
	-Sex/fertility (37,44,51)		-How to communicate effectively with health professionals(54)
	-How to communicate effectively with health professionals(51)		
**How**	- Empathy, competence, honesty, patience (39,46,33,35,34,40,49,52)		- Empathy, competence, honesty, patience (32,44)
	- Communication language (34,49,51,52,43)		- Communication language (29)
	- Communication type (44)		- Communication type (31,32)
	- Delivery manner (33,50,48,30)		- Delivery manner (31,33)
	- Personalised communication (35,40,47)		- Different communication needs at different stages (54)
	- Different communication needs at different stages (34)		- Communication channels (42)
	- Communication channels(49,42)		
**When**	- As early as possible (29,50,30)		- Discuss patient prognosis early (32)
	- Close to death ,obtain consent, based on emotional state(33)		- Close to death ,obtain consent, based on emotional state(33)
**Preferences**	- Gender (52);	- Age (49)	- Work status, caregiver type (54)
	- Education level (40,37)	- Cross-cultural (40,33)	
	- Geographic factors (36)	- Cancer site (36,38,52)	
	- Medical condition (50)		

*The number in brackets indicates the related reference literature number cited in the manuscript.
